# Immunosenescence and inflammaging: Mechanisms and modulation through diet and lifestyle

**DOI:** 10.3389/fimmu.2025.1708280

**Published:** 2025-12-04

**Authors:** Ludmila Müller, Svetlana Di Benedetto

**Affiliations:** Max Planck Institute for Human Development, Center for Lifespan Psychology, Berlin, Germany

**Keywords:** aging, immunosenescence, inflammaging, crosstalk, dietary and lifestyle modulation

## Abstract

Aging is associated with profound alterations in the immune system, characterized by immunosenescence and inflammaging, which together compromise host defense, promote chronic low-grade inflammation, and contribute to the development of age-related diseases. Immunosenescence involves thymic involution, hematopoietic stem cell skewing, accumulation of senescent immune cells, and impaired adaptive and innate responses. Inflammaging arises from persistent activation of innate immune pathways, senescence-associated secretory phenotype (SASP) signaling, metabolic dysregulation, and age-related alterations in the gut microbiome. These processes are interconnected through feedback loops and network-level interactions among immune, metabolic, and microbial systems, creating a self-perpetuating cycle of immune dysfunction and systemic inflammation. Emerging evidence indicates that immunosenescence and inflammaging can be modulated through integrative strategies that combine nutrition, microbiome modulation, and lifestyle interventions to sustain immune resilience across the lifespan. Nutrient-specific strategies, including polyphenols, omega-3 fatty acids, and micronutrients, regulate oxidative stress, cytokine signaling, and immune cell metabolism. Holistic dietary patterns such as the Mediterranean diet, caloric restriction, and microbiome-supportive diets enhance gut barrier integrity, modulate systemic inflammation, and improve adaptive immunity. Lifestyle factors, including regular physical activity, adequate sleep, and stress reduction, further support immune resilience. Personalized nutrition and lifestyle strategies, guided by immunobiological profiling, enable tailored approaches to mitigate immune aging. Collectively, these insights highlight a multidimensional framework for understanding and modulating immunosenescence and inflammaging. Integrating dietary, lifestyle, and pharmacological strategies offers a promising path toward enhancing immune function, reducing chronic inflammation, and promoting healthy longevity.

## 1 Introduction

**Figure 1:**
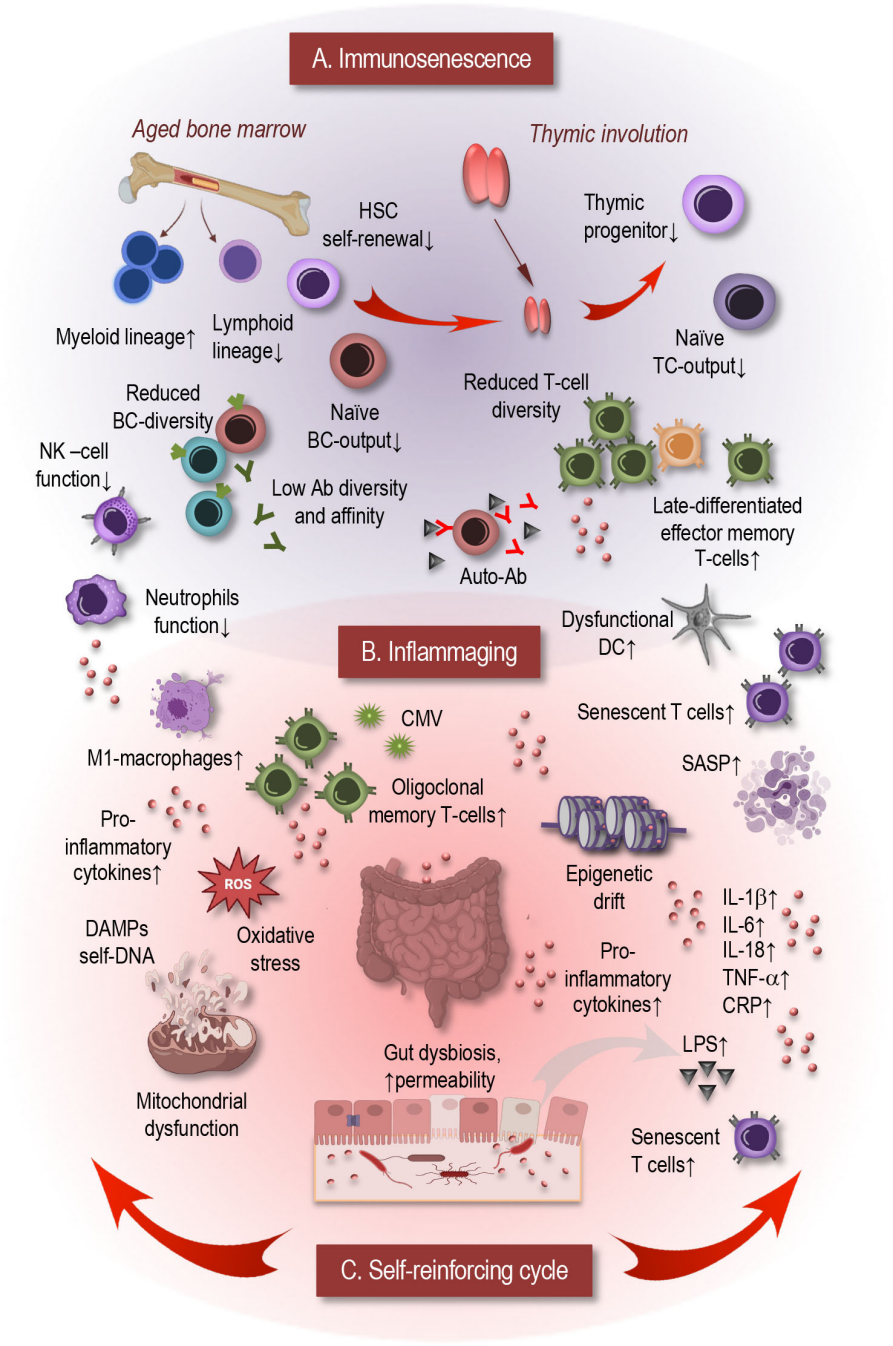
This simplified scheme illustrates dynamic self-reinforcing interactions between immunosenescence and inflammaging. **(A)** Immunosenescence. Aging of hematopoietic stem cells (HSCs) represents a central upstream mechanism driving immunosenescence. HSCs undergo functional decline with reduced self-renewal capacity and lineage skewing toward myeloid differentiation at the expense of lymphoid progenitors. This results in diminished output of naïve T and B cells, expansion of myeloid-derived cells, and impaired function of neutrophils, dendritic cells, NK cells, and macrophages. Thymic involution further reduces naïve T-cell output, restricting T-cell receptor repertoire diversity and favoring accumulation of late-differentiated, senescent-like T cells. In parallel, the B-cell compartment shows decreased naïve B-cell generation and impaired class-switch recombination, leading to reduced antibody diversity and affinity, while autoreactive B-cell clones accumulate, predisposing to autoimmunity. **(B)** Inflammaging. Immunosenescence promotes accumulation of senescent immune cells that secrete pro-inflammatory mediators via the SASP, driving chronic low-grade inflammation. Additional contributors include chronic viral infections such as CMV, which sustain oligoclonal expansion of memory T cells and continuous cytokine release. The aging microbiome, characterized by dysbiosis and reduced microbial diversity, compromises gut barrier integrity, increasing microbial translocation (e.g., LPS) and systemic immune activation. Cellular-intrinsic factors, including DNA damage, mitochondrial dysfunction, and oxidative stress, lead to release of DAMPs, further activating innate immune pathways. **(C)** Self-reinforcing cycle. Immunosenescence and inflammaging are mutually reinforcing processes: age-associated immune dysfunction fuels chronic inflammation, which in turn accelerates immune aging, establishing a self-perpetuating cycle. HSC, hematopoietic stem cell; TC, T cell; BC, B cell; NK, natural killer cell; Ab, antibodies; DC, dendritic cell; CMV, Cytomegalovirus; SASP, senescence-associated secretory phenotype; DAMP, damage-associated molecular pattern; DNA, deoxyribonucleic acid; ROS, reactive oxygen species; IL, interleukin; TNF, tumor necrosis factor; CRP, C-reactive protein; LPS, lipopolysaccharide.

Aging fundamentally reshapes the immune system, impairing its ability to respond to new threats while simultaneously giving rise to persistent, sterile inflammation (1). This dual phenomenon—immunosenescence (**Figure 1A**) on one hand, and inflammaging (**Figure 1B**) on the other—is central to understanding age-related immune decline (2).

Immunosenescence involves a progressive deterioration of both adaptive and innate immunity. Thymic involution substantially reduces naïve T-cell output, shrinking T-cell receptor diversity and skewing the CD4^+^/CD8^+^ ratio (3). Concurrently, the accumulation of senescent, exhausted, or memory T and B cells, along with diminished NK-cell cytotoxicity and antigen-presenting cell efficacy, undermines responsiveness to infections and vaccinations (2). Adaptive immune cells exhibit altered metabolic profiles—impaired oxidative phosphorylation, reduced SIRT1 activity, and telomeric attrition—all hallmarks of cellular aging (4–7).

Parallel to immune decline, inflammaging manifests as chronic, low-grade systemic inflammation marked by elevated IL-6, IL-1β, TNF-α, IL-8 and C-reactive protein (CRP) in the absence of acute infection (8–10). Aging tissues accumulate senescent cells that secrete a complex mixture of inflammatory cytokines, chemokines, growth factors, and matrix-degrading proteases—a profile collectively termed the SASP (6). The chronic inflammation that defines inflammaging arises from multiple interconnected mechanisms. Dysfunctional autophagy and mitophagy lead to the accumulation of damaged organelles and oxidative stress, which in turn release damage-associated molecular patterns (DAMPs) such as mitochondrial DNA. These endogenous ligands activate innate immune sensors—TLRs and NLRs—triggering inflammasome assembly and cytokine release, thereby sustaining systemic inflammation over time (2, 6, 7, 11).

Importantly, immunosenescence and inflammaging exist in a harmful feedback loop. Inflammatory SASP factors impair hematopoietic stem cell renewal and naïve lymphocyte production, restricting immune reconstitution. Meanwhile, chronic inflammation fosters expansion of immunosuppressive cell populations—such as regulatory T cells, myeloid-derived suppressor cells (MDSCs), and M2 macrophages—that further dampen adaptive immune responses and promote tissue dysfunction (12–14). The functional consequences are profound: elderly individuals become more susceptible to infections (e.g., influenza, COVID-19), show reduced vaccine efficacy, and face enhanced risk of cancer, cardiovascular, neurodegenerative, and metabolic conditions (5, 8, 15, 16).

Diet and lifestyle exert powerful influence over immune aging through their impact on microbial communities, mucosal health, and metabolic and signaling pathways. High-fiber, polyphenol-rich, and omega-3–enriched diets support microbiota eubiosis, enhance integrity of the intestinal barrier, reduce inflammatory tone, and promote the production of immunometabolic regulators like SCFAs that support T-cell and monocyte function (17). Physical activity, caloric restriction, and specific nutritional interventions have been linked to modulation of immunosenescence markers, maintenance of repertoire diversity, and reduced inflammatory mediators in older adults (18).

This mini-review integrates mechanistic insights from immunosenescence and inflammaging with evidence-based interventions involving diet, microbiota modulation, and lifestyle strategies. By synthesizing current efficacy and effectiveness data, we aim to explore how nutritional, microbial, metabolic, and behavioral approaches can collectively support immune resilience and promote healthy aging.

## 2 Mechanisms of immunosenescence

Immunosenescence refers to the progressive remodeling of immune functions with advancing age, encompassing both the innate and adaptive branches of the immune system. Rather than a uniform decline, it represents a complex reorganization in which selective functions are lost, compensatory adaptations arise, and maladaptive processes emerge, ultimately compromising host defense and immune homeostasis (19–21).

Aging of the hematopoietic stem cell (HSC) compartment represents a central upstream mechanism driving immunosenescence (**Figure 1A**). With advancing age, HSCs undergo functional decline characterized by reduced self-renewal capacity, accumulation of DNA damage, epigenetic alterations, and metabolic dysfunction (22). Importantly, aged HSCs exhibit lineage skewing, with a preferential differentiation toward the myeloid lineage at the expense of lymphoid progenitors. This results in a diminished output of naïve T and B cells and a concomitant expansion of myeloid-derived cells, thereby contributing to both immunosenescence and inflammaging. These changes in hematopoietic stem and progenitor cells establish an early bottleneck that underlies many of the downstream immune alterations observed in older individuals (22–24).

The innate immune system undergoes substantial functional decline during aging. Neutrophils display impaired chemotaxis, phagocytosis, and diminished generation of reactive oxygen species (ROS), resulting in decreased antimicrobial capacity. Dendritic cells show reduced antigen uptake and migration, which impairs efficient T-cell priming. Natural killer cells undergo numerical and functional shifts, with an expansion of the mature CD56^dim^ cytotoxic subset but reduced cytokine production and proliferative potential, thereby weakening immunosurveillance against infections and tumors. Similarly, macrophages exhibit reduced phagocytic activity, impaired antigen presentation, and altered polarization between pro- and anti-inflammatory states, which contributes to the establishment of chronic low-grade inflammation (25–27).

Alterations within the adaptive immune system are even more striking. Thymic involution markedly reduces the output of naïve T cells, leading to a contraction of the T-cell receptor (TCR) repertoire and a reduced ability to respond to novel antigens (28). As a consequence, peripheral T-cell homeostasis becomes increasingly dependent on clonal expansion of pre-existing memory cells. This leads to the accumulation of late-differentiated and senescent-like T cells, including CD28^−^ T cells and CD45RA^+^ effector memory cells (TEMRA), which are characterized by diminished proliferative potential but a strong pro-inflammatory profile (29). CD4^+^ T-cell differentiation is skewed, with an increased Th17 polarization and reduced T follicular helper activity, resulting in impaired B-cell help and reduced vaccine responsiveness (30). The B-cell compartment is likewise affected, with a decline in the generation of naïve B cells and defects in class-switch recombination, leading to lower antibody diversity and affinity. At the same time, autoreactive B-cell clones accumulate, which may contribute to the higher prevalence of autoimmunity in the elderly (31, 32).

Several intrinsic molecular and cellular mechanisms further exacerbate these age-associated changes. Telomere attrition limits the proliferative potential of lymphocytes, particularly T cells, thereby contributing to replicative senescence (33). Epigenetic drift, involving alterations in DNA methylation and histone modifications, reshapes the transcriptional landscape of immune cells and affects their differentiation and activation potential (34). Mitochondrial dysfunction further exacerbates immune decline by impairing bioenergetics and increasing ROS production, thereby fueling both cellular senescence and the chronic pro-inflammatory state of inflammaging (35). The accumulation of senescent immune cells, which often express inhibitory receptors such as PD-1 and KLRG1 and secrete pro-inflammatory mediators through the senescence-associated secretory phenotype, creates a vicious cycle that further drives immune dysfunction (36, 37).

Together, these alterations result in a reduced capacity to mount protective responses, impaired immunological memory, and a simultaneous increase in pro-inflammatory activity. Thus, immunosenescence not only undermines protective immunity but also sets the stage for chronic inflammation, or inflammaging, the mechanisms of which will be explored in the following section.

## 3 Mechanisms of inflammaging

Inflammaging (**Figure 1B**) is defined as the chronic, low-grade, systemic inflammation that develops with age and represents a central hallmark of immunosenescence (1). Unlike acute inflammation, which is a tightly regulated response to infection or tissue injury, inflammaging persists over time, even in the absence of overt pathology, and contributes to a broad spectrum of age-related diseases including cardiovascular disease, type 2 diabetes, neurodegeneration, and cancer (9).

Several sources contribute to inflammaging. Senescent cells accumulate in multiple tissues with age and adopt a SASP, characterized by the secretion of pro-inflammatory cytokines (e.g., IL-6, IL-1β, TNF-α), chemokines, proteases, and growth factors. These mediators promote local and systemic inflammation and can induce senescence in neighboring cells, creating a feed-forward loop that amplifies tissue dysfunction (38). In the immune system, accumulation of late-differentiated T cells and myeloid-biased cells from aged HSCs also contributes to a pro-inflammatory environment, as these cells produce higher levels of inflammatory cytokines and respond aberrantly to stimuli (19).

Chronic infections, such as cytomegalovirus (CMV), serve as another driver of inflammaging. Persistent viral antigenic stimulation leads to expansion of oligoclonal memory T cells and continuous low-level cytokine production, further maintaining systemic inflammation (13, 39, 40). Similarly, lifelong exposure to environmental antigens and subclinical tissue damage results in repeated innate immune activation, generating pro-inflammatory signals that do not resolve efficiently in older adults (7, 13).

The aging microbiome also plays a pivotal role. Dysbiosis, often characterized by reduced microbial diversity and a loss of beneficial commensals, compromises gut barrier integrity, increases intestinal permeability, and allows translocation of microbial products such as lipopolysaccharide (LPS) into the circulation, thereby activating systemic inflammation (41). Metabolic dysfunction, including insulin resistance, adipose tissue expansion, and ectopic lipid accumulation, further exacerbates inflammaging through production of adipokines and activation of pro-inflammatory signaling pathways such as NF-κB and NLRP3 inflammasomes (42).

Finally, cell-intrinsic factors amplify the inflammatory milieu. Accumulation of DNA damage, mitochondrial dysfunction, impaired autophagy, and oxidative stress in both immune and non-immune cells promote release of DAMPs, which trigger innate immune receptors and perpetuate cytokine secretion (43). The convergence of these intrinsic and extrinsic pathways establishes a persistent pro-inflammatory state that, in combination with immunosenescence, compromises immune competence while driving tissue aging and pathology.

Taken together, inflammaging represents both a consequence and a driver of immune aging: senescent and dysregulated immune cells propagate chronic inflammation, which in turn accelerates tissue dysfunction and reinforces immune decline (2, 13, 40). Understanding these interconnected mechanisms is critical for developing interventions aimed at reducing age-related inflammation and improving immune resilience in older adults.

## 4 Crosstalk between immunosenescence and inflammaging

As discussed in previous sections, immunosenescence and inflammaging are deeply interconnected processes that mutually reinforce each other (**Figure 1C**), creating a vicious cycle of immune dysfunction and chronic low-grade inflammation. Age-related impairments in immune cell function, including reduced pathogen recognition, defective antigen presentation, and diminished adaptive responses, lead to persistent microbial and cellular stress signals. These unresolved stimuli drive continuous activation of innate immune cells, resulting in sustained production of pro-inflammatory cytokines such as IL-6, TNF-α, and IL-1β, which in turn exacerbate systemic inflammation (9, 19). Senescent immune and non-immune cells further amplify this feedback loop. Cells that have undergone replicative or stress-induced senescence secrete the SASP, comprising pro-inflammatory cytokines, chemokines, and proteases, which promote inflammation locally and systemically (38).

From a systems perspective, immune, metabolic, and microbiome networks interact dynamically to regulate inflammation and immune competence. Metabolic alterations in immune cells, such as mitochondrial dysfunction or impaired glycolysis, enhance pro-inflammatory signaling and limit effective immune responses (35). Concurrently, age-associated changes in the gut microbiome increase intestinal permeability and allow translocation of microbial products into the circulation, which stimulates innate immune receptors and promotes systemic inflammation (41). Adipose tissue dysfunction in aging contributes additional pro-inflammatory signals, linking metabolic dysregulation directly to immunosenescence and inflammaging (35, 42, 44).

Thus, the interplay between immune dysfunction, senescent cells, and metabolic-microbiome networks forms a complex, self-reinforcing system. Understanding these interconnected pathways is essential for designing interventions aimed at breaking this cycle and restoring immune resilience in older adults.

## 5 Modulation through overall dietary patterns and nutrient-specific components

**Figure 2:**
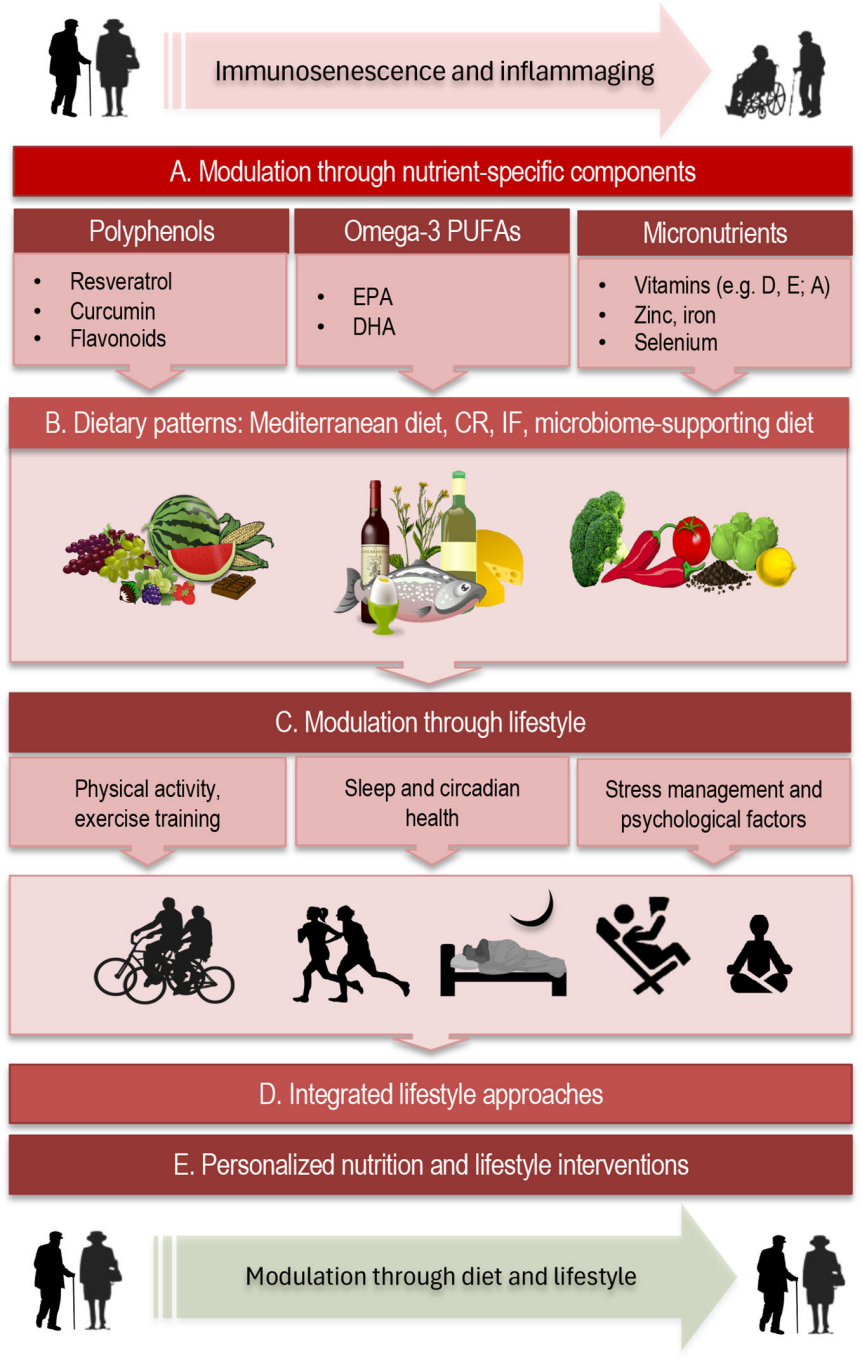
Mechanisms and modulation of immune aging through diet and lifestyle. **(A)** Nutrient-specific components. Polyphenols (e.g., resveratrol, curcumin, flavonoids) exert antioxidant and anti-inflammatory effects via multiple, complementary pathways directly relevant to immune aging. Omega-3 PUFAs, particularly EPA and DHA, have well-established immunomodulatory properties. Micronutrients such as vitamins D, E, and A, as well as zinc and selenium, are essential for maintaining immune homeostasis and resilience. **(B)** Dietary patterns. Adherence to overall dietary patterns provides synergistic benefits for immune health beyond single nutrients. The Mediterranean diet, characterized by high intake of fruits, vegetables, legumes, whole grains, nuts, olive oil, and moderate fish consumption, is strongly linked to anti-inflammatory and pro-longevity effects. Caloric restriction (CR) and intermittent fasting (IF) have been shown to modulate immunosenescence and inflammaging. Plant-based and microbiome-supporting diets further promote immune health by reshaping gut microbial composition and function. **(C)** Lifestyle factors. Physical activity, restorative sleep, and effective stress management are central regulators of immune function across the lifespan. **(D)** Integrated lifestyle approaches. Combining multiple dietary and lifestyle interventions enhances immune resilience, providing additive or synergistic effects that surpass single-factor approaches. **(E)** Personalized strategies. Precision nutrition and tailored lifestyle approaches are emerging as translational pathways to mitigate immunosenescence, offering a framework for healthier immune aging and a systems-level integration of interventions. PUFA, polyunsaturated fatty acids; EPA, eicosapentaenoic acid; DHA, docosahexaenoic acid; CR, caloric restriction; IF, intermittent fasting.

Dietary interventions have emerged as powerful modulators of immune aging and inflammaging, influencing immune cell function, systemic inflammation, and metabolic homeostasis. Both nutrient-specific components (**Figure 2A**) and overall dietary patterns (**Figure 2B**) contribute to shaping immune resilience in older adults. The discussion that follows first addresses nutrient-specific effects, examining how individual dietary components—from polyphenols and fatty acids to essential vitamins and trace minerals—interact with immune and metabolic pathways to counteract immunosenescence and inflammaging.

### 5.1 Nutrient-specific effects of polyphenols

Polyphenols, including resveratrol, curcumin, and flavonoids, exhibit antioxidant and anti-inflammatory effects in experimental models, acting on multiple complementary pathways relevant to immune aging (45). While their direct bioactivity in humans may be limited by low systemic bioavailability, polyphenols can also modulate immune function indirectly via gut microbiota–derived metabolites (46). Mechanistically, many polyphenols suppress established inflammatory signaling — notably NF-κB — and inhibit activation of the NLRP3 inflammasome, actions that reduce IL-1β/IL-18 release and attenuate the SASP. These anti-inflammatory effects are accompanied by antioxidant actions (reduced ROS) and epigenetic modulation that together temper chronic inflammatory signaling (45, 47).

Beyond inflammation suppression, several polyphenols promote cellular programs that improve immune cell fitness. Resveratrol and related stilbenes stimulate SIRT1-dependent pathways and PGC-1α signaling that favor mitochondrial biogenesis and improved bioenergetics in lymphocytes; resveratrol also engages AMPK/SIRT1 axes that can blunt inflammasome activation. These metabolic effects help sustain T-cell function and reduce inflammatory phenotypes in aging models (45, 48).

Curcumin and selected flavonoids further enhance proteostasis and autophagy, supporting removal of damaged organelles (including dysfunctional mitochondria) and limiting inflammasome-driven pyroptosis. Curcumin has been repeatedly shown in recent mechanistic studies to downregulate NLRP3 activation, induce autophagy, and up-regulate SIRT1 in relevant cell and animal models, linking these pathways to reduced inflammaging (49).

Animal studies have provided compelling evidence that polyphenols modulate both innate and adaptive immunity. In mice, resveratrol supplementation has been shown to enhance T-cell proliferation, increase NK-cell activity, and reduce pro-inflammatory cytokine production, such as IL-6 and TNF-α, during aging or in models of chronic inflammation (50, 51). Curcumin and flavonoid-rich diets have been associated with reduced oxidative stress, improved macrophage function, and modulation of signaling pathways such as NF-κB and Nrf2, which are central to immune regulation (52). These studies suggest that polyphenols can counteract hallmarks of immunosenescence, including chronic low-grade inflammation and diminished adaptive immune responses (52, 53).

Recent studies have further elucidated the immunomodulatory effects of polyphenols in animals. For instance, dietary supplementation with tea polyphenols has been shown to enhance antioxidant capacity, improve gut microbiota composition, and bolster immune function in various animal models. These findings underscore the potential of polyphenols as functional ingredients in animal nutrition to promote health and disease resistance (54).

Human studies, although more limited, further support the potential immunomodulatory effects of polyphenols (55). Clinical trials have demonstrated that supplementation with flavonoid-rich foods, such as green tea catechins or cocoa, can enhance NK-cell activity, improve antibody responses to vaccines, and reduce markers of systemic inflammation in older adults (45, 55). Resveratrol supplementation has been reported to lower circulating inflammatory markers, including CRP and IL-6, and improve T-cell proliferative capacity. A recent study highlighted the impact of a polyphenol-rich supplement on immune cell epigenetics in dyslipidemic humans, suggesting that polyphenols may influence immune function through epigenetic mechanisms (56).

However, as mentioned above, many human studies are constrained by the low bioavailability of polyphenols, which limits the translation of effects observed *in vitro* and in animal models. Interestingly, some immunomodulatory effects may be mediated indirectly through the gut microbiota, which metabolizes poorly absorbed polyphenols into bioactive compounds that can influence systemic immune responses (46, 57).

Thus, clinical and translational evidence is growing but heterogeneous: recent reviews and trials report that polyphenol-rich dietary patterns or defined supplements can lower inflammatory biomarkers, improve aspects of immune function, and in some settings correlate with better vaccine or infection-related outcomes in older adults; however, effect sizes vary with compound, dose, formulation, and study population, and larger, well-controlled trials are still needed to define robust clinical recommendations (45).

### 5.2 Omega-3 polyunsaturated fatty acids

Omega-3 PUFAs, particularly eicosapentaenoic acid (EPA) and docosahexaenoic acid (DHA), are essential fatty acids with well-established anti-inflammatory and immunomodulatory properties. Mechanistically, omega-3 PUFAs modulate inflammatory pathways by reducing the production of pro-inflammatory cytokines and promoting anti-inflammatory mediators. They achieve this in part through activation of peroxisome proliferator-activated receptors (PPARs) and inhibition of NF-κB signaling, thereby dampening systemic inflammatory responses. In addition, omega-3 PUFAs serve as precursors for specialized pro-resolving mediators (SPMs), such as resolvins and protectins, which actively promote the resolution of inflammation and tissue repair (58).

These fatty acids also influence immune cell function directly. By improving membrane fluidity and receptor signaling, omega-3 PUFAs enhance the activity of macrophages and T lymphocytes, optimizing both innate and adaptive immune responses (59). Higher levels of omega-3 PUFAs have been associated with a reduction in biological age, as measured by epigenetic clocks, suggesting a potential role in mitigating age-related cellular decline (60). Regular intake of EPA and DHA has also been linked to a lower risk of chronic diseases prevalent in aging populations, including cardiovascular and neurodegenerative disorders, likely due to their anti-inflammatory effects (61–63). In older adults, omega-3 supplementation has been shown to support muscle mass and function, counteracting sarcopenia and frailty (29).

From a translational perspective, clinical studies indicate that omega-3 supplementation can reduce markers of systemic inflammation, such as CRP, in elderly populations (64). Incorporating omega-3-rich foods, including fatty fish such as salmon and mackerel, as well as plant sources like flaxseeds and walnuts, can provide these beneficial fatty acids. While optimal dosage may vary depending on individual factors, studies suggest that daily intake of 1–2 grams of combined EPA and DHA effectively reduces inflammation and supports immune function (65).

### 5.3 Micronutrients

Micronutrients, such as vitamin D, zinc, and selenium are critical for immune homeostasis and resilience. Recent studies increasingly highlight the importance of specific vitamins and trace minerals in preserving immune competence during aging.

Vitamin D modulates both innate and adaptive immunity by promoting antimicrobial peptide production, enhancing macrophage and dendritic cell function, and regulating T-cell differentiation toward a more anti-inflammatory profile, including increased regulatory T-cell activity and reduced Th1/Th17 responses (66).

A randomized clinical trial involving older adults with vitamin D deficiency found that daily supplementation with vitamin D and N-acetylcysteine over eight weeks significantly reduced expression of senescence markers (including p16), lowered levels of IL-6 and TNF-α, and decreased activity of senescence-associated β-galactosidase in peripheral blood mononuclear cells. This suggests that correcting vitamin D deficiency can directly ameliorate immunosenescence and dampen systemic inflammation (67).

Another recent trial tested high-dose vitamin D_3_ replacement (6,400 IU/day for 14 weeks) in older adults who were insufficient in vitamin D. The intervention enhanced antigen-specific cutaneous immune responses to varicella zoster virus challenge and was associated with reduced infiltration of inflammatory monocytes and increased recruitment of T cells in the skin. The results imply that sufficient vitamin D status supports more effective immune activation in aging tissues (68).

Animal and human studies show that vitamin E deficiency impairs humoral and cell-mediated immunity, whereas supplementation above recommended levels enhances immune responses and resistance to infections. Vitamin E, a lipid-soluble antioxidant, protects cell membranes from oxidative damage and modulates T-cell function through regulation of membrane integrity and signal transduction. Supplementation with vitamin E has been shown to enhance T-cell–mediated responses and lower markers of oxidative stress in older adults (69).

Meta-analytic and narrative reviews have also shown that in respiratory infections—especially COVID-19—micronutrients like vitamins D, E, A, iron, zinc, selenium, and magnesium may influence disease risk and severity. These nutrients appear to act through bolstering mucosal immunity, antioxidant defenses, and reducing inflammatory damage. Populations with poor micronutrient status tend to exhibit worse outcomes, underscoring the translational potential of correcting deficiencies in older age (70).

Zinc is a vital trace element integral to numerous physiological processes, including immune function, DNA synthesis, and cell division. It serves as a structural and catalytic cofactor for over 300 enzymes and transcription factors, underscoring its importance in cellular metabolism and immune responses (71, 72). Age-related zinc deficiency has been implicated in the decline of both innate and adaptive immunity. This deficiency can lead to dysregulation of NF-κB signaling and reduced thymulin activity, a thymic hormone essential for T-cell differentiation (73, 74). Conversely, maintaining adequate zinc levels supports thymic output, enhances T-cell responses, and supports antiviral defenses. Supplementation has shown promise in reversing age-related thymic involution and improving immune function, particularly in older adults (75).

Selenium is an essential micronutrient that plays a critical role in maintaining redox balance, regulating immune responses, and preserving metabolic homeostasis. Incorporated into selenoproteins such as glutathione peroxidases and thioredoxin reductases, selenium mitigates oxidative stress and modulates redox-sensitive signaling pathways that influence both innate and adaptive immunity. Selenium deficiency has been linked to increased susceptibility to infections and an exaggerated pro-inflammatory state characteristic of inflammaging (76, 77). Collectively, these micronutrients contribute to maintaining redox balance, immune competence, and resilience against age-related inflammation.

There is emerging evidence that combined nutritional supplementation can influence biological aging metrics. A study of healthy older adults with elevated epigenetic age demonstrated that a multicomponent nutritional supplement containing vitamin B3, C, D, omega-3, resveratrol, olive fruit phenols, and astaxanthin partially reduced DNA methylation age only in those with high baseline epigenetic age, along with reductions in inflammatory markers. This indicates both the feasibility and limitations of micronutrient or mixed nutrient approaches in slowing inflammaging in susceptible individuals (78).

Taken together, these findings suggest that maintaining adequate status of vitamin D, zinc, selenium, and the antioxidant vitamins is more than just supportive—it may modulate the core processes of immune aging. However, effects depend on baseline deficiency, dose, duration, and individual variation, including genetic and microbiome influences.

### 5.4 Dietary patterns

Adherence to healthy dietary patterns (**Figure 2B**), such as the Mediterranean diet, in contrast to Western or pro-inflammatory diets, provides synergistic benefits for immune health beyond those of individual nutrients. The Mediterranean diet, characterized by high consumption of fruits, vegetables, legumes, whole grains, nuts, and olive oil alongside moderate intake of fish, is strongly associated with anti-inflammatory and pro-longevity effects. Beyond its well-documented impact on cardiovascular and metabolic health, adherence to this dietary pattern improves T-cell functionality, reduces systemic inflammatory markers such as CRP and IL-6, and fosters a more diverse and resilient gut microbiome (79, 80).

Recent human and animal studies continue to illuminate how caloric restriction (CR) and intermittent fasting (IF) can modulate immunosenescence and inflammaging. In healthy humans undergoing moderate caloric restriction (~14% over two years), there was evidence of increased thymic output, reduced fat infiltration in the thymus, and transcriptional changes in adipose tissue consistent with improved mitochondrial bioenergetics and reduced inflammation (81). Animal models also support these findings: long-term CR in mice has been shown to substantially preserve naïve CD4^+^ and CD8^+^ T-cell populations, reduce markers of T-cell exhaustion (such as PD-1, TIM-3, KLRG1), and maintain NK/NKT cell frequencies compared to ad libitum feeding (82).

Intermittent fasting shows complementary benefits. Studies have shown that fasting schedules activate nutrient-sensing pathways such as AMP-activated protein kinase (AMPK) and suppress the mammalian target of rapamycin (mTOR) pathway. These shifts favor autophagy, improve mitochondrial function, and reduce oxidative stress (83). In murine models of rheumatoid arthritis, IF reduced joint inflammation and damage, modulated gut microbiota composition, and lowered expression of inflammatory mediators (84). More broadly, reviews of IF interventions note improvements in systemic inflammatory markers, better metabolic flexibility in immune cells, and attenuation of NF-κB–mediated signaling (85).

Together, these findings suggest that CR and IF do more than merely reduce caloric load: they trigger metabolic rewiring in immune cells (improving mitochondrial efficiency and autophagy), preserve naïve T-cell pools, delay or reduce T-cell exhaustion, and lower systemic pro-inflammatory cytokine levels. However, human data remain more limited and heterogenous, with questions about optimal duration, degree of restriction, and individual variability needing further clarification.

Plant-based or microbiome-supporting diets exert broad immunomodulatory effects by reshaping gut microbial composition and function. A central mechanism involves the production of short-chain fatty acids (SCFAs)—including butyrate, acetate, and propionate—generated through the fermentation of dietary fibers by commensal bacteria. SCFAs not only serve as a primary energy source for colonocytes and preserve epithelial barrier integrity, but also act as signaling molecules linking microbial metabolism to host immunity. Butyrate, in particular, promotes the differentiation and stability of regulatory T cells, suppresses pro-inflammatory cytokine production, and modulates gene expression via histone deacetylase inhibition, thereby integrating dietary and microbial signals into host epigenetic regulation (86–88).

Beyond local intestinal effects, SCFAs exert systemic actions by influencing hematopoietic stem cell function, enhancing bone marrow lymphopoiesis, and limiting the expansion of pro-inflammatory myeloid cells. This systemic impact is particularly relevant in the context of immune aging, where HSC skewing toward myelopoiesis contributes to inflammaging (89–91).

Together, these findings highlight the gut–immune–metabolic axis as a central target for interventions aimed at mitigating immunosenescence and inflammaging. By supporting gut barrier integrity, enhancing SCFA-mediated regulatory pathways, and limiting microbial translocation of pro-inflammatory molecules, plant-based and microbiome-directed diets represent powerful, evidence-based strategies to preserve immune resilience and promote healthy aging.

Overall, both nutrient-specific interventions and holistic dietary patterns exert complementary effects, influencing immune metabolism, reducing chronic inflammation, and supporting adaptive and innate immune responses. These findings underscore the importance of diet as a modifiable factor to counteract immunosenescence and inflammaging.

## 6 Modulation through lifestyle

Lifestyle factors (**Figure 2C**), including physical activity, sleep, and stress management, play a central role in shaping immune health across the lifespan. Integrated lifestyle interventions influence immune cell metabolism, inflammatory signaling, and host–microbiome interactions, collectively enhancing immune resilience in older adults. Tailoring these strategies to individual biological and lifestyle contexts can further optimize their impact, targeting specific vulnerabilities and reinforcing the network-level connections among immune, metabolic, and microbial systems. In the following subsections, we discuss key lifestyle domains—exercise, sleep, stress reduction, and multi-domain interventions—and their roles in modulating immunosenescence and inflammaging.

### 6.1 Physical activity

Recent trials and cohort studies continue to provide robust evidence that regular moderate exercise exerts multifaceted beneficial effects on both innate and adaptive immunity, particularly in older populations. Cross-sectional and interventional studies likewise show that physically active older people maintain better NK-cell activity, lower proportions of pro-inflammatory monocyte subsets, and more efficient neutrophil migration compared to less active peers (92). In addition, recent work indicates that endurance exercise interventions increase the numbers of naïve T-helper and cytotoxic T cells while reducing effector and memory T-cell subsets (including TEMRA) in older adults—patterns consistent with preservation of a more youthful adaptive immune composition (93). In older adults at risk of type 2 diabetes, a ten-week high-intensity interval training (HIIT) protocol was shown to rejuvenate neutrophil functions — enhancing chemotaxis, phagocytosis, and improving mitochondrial bioenergetics — paralleling improvements in cardiorespiratory fitness and glucose control (94).

Though direct data in humans remains limited, these studies suggest that regular physical activity reduces systemic inflammation - lowering markers like CRP, IL-6, TNF-α – while promoting the production of anti-inflammatory cytokines like IL-10. Several human studies show that maintaining regular physical activity is associated with slower decline in thymic output or higher levels of recent thymic emigrants compared with less active peers, as assessed by markers such as PTK7-positive T cells and serum thymoprotective cytokines (95, 96). Moreover, recent studies demonstrate that even simple progressive resistance training in older adults can enhance physical performance, decrease systemic inflammatory mediators associated with the senescence-associated secretory phenotype (SASP), and reduce markers of T-cell senescence and immune dysfunction (97).

Together, the accumulating evidence indicates that regular exercise acts through multiple layers—innate immune cell function, adaptive T-cell subset preservation, inflammatory milieu modulation, and possibly thymic support—to enhance immunosurveillance, reduce inflammaging, and slow immune aging curves.

### 6.2 Sleep and circadian health

Adequate sleep and close alignment with circadian rhythms are essential for preserving immune competence, particularly during aging. Sleep deprivation or chronic disruption elevates pro-inflammatory cytokines such as IL-6 and TNF-α, diminishes NK-cell cytotoxicity, weakens T-cell proliferation, and reduces the magnitude and durability of antibody responses following vaccination (98, 99). In older adults, better sleep quality has been associated with more persistent antibody titers to common infectious agents, highlighting the role of sleep in maintaining immune memory (100).

At the stem cell level, experimental models suggest that poor sleep perturbs hematopoietic stem and progenitor cell epigenetics, enhances proliferation, and biases differentiation toward myeloid lineages, thereby promoting inflammaging (101). Furthermore, sleep actively promotes lymphocyte trafficking: under normal sleep, compared with nocturnal wakefulness, T-cell subsets show enhanced migration toward the chemokine CCL19, supporting efficient immunosurveillance (102).

Recent studies increasingly point toward melatonin as a promising agent in modulating immune aging. A recent narrative review highlights that melatonin levels decline with age and that sleep deprivation (which suppresses melatonin) correlates with elevated pro-inflammatory cytokines, increased oxidative stress, and reduced NK-cell and CD4^+^ T-cell activity, suggesting that melatonin plays a mediatory role in linking circadian disruption to immune dysregulation (103).

In aged mice, melatonin treatment was found to enhance NK-cell function by increasing proliferation, degranulation, and IFN-γ secretion (104). Moreover, melatonin treatment in aged mice was shown to restore rhythmic expression of clock genes disrupted by aging, counteracting activation of the NLRP3 inflammasome in cardiac tissue and ameliorating age-associated chronodisruption (105).

Other animal studies show that continuous melatonin administration reverses age-related thymic involution, increases thymus size and thymocyte number, and enhances peripheral immune responses, including NK activity and mitogen responsiveness, even when treatment begins late in life (106). Further, meta-analytic evidence in human trials supports that exogenous melatonin can significantly reduce levels of IL-1, IL-6, IL-8, and TNF in various inflammatory conditions, though effects on CRP are mixed (107).

These insights place adequate sleep and circadian health at the center of immune resilience, with melatonin serving as a key link between biological timing and healthy aging. Together, these findings underscore that melatonin’s immunoregulatory actions are closely tied to circadian health, highlighting the importance of maintaining robust circadian rhythms in sustaining immune resilience and counteracting age-related inflammation.

### 6.3 Stress management and psychosocial factors

Chronic psychological stress accelerates immune aging through dysregulation of the hypothalamic-pituitary-adrenal (HPA) axis, glucocorticoid resistance, and elevated systemic inflammation. For example, older adults exposed to prolonged loneliness or high stress show impaired glucocorticoid receptor sensitivity and elevated inflammatory gene transcription, which mirror features of accelerated immunosenescence. Recent randomized controlled trials in older populations demonstrate that mind-body interventions—such as Mindfulness-Based Stress Reduction (MBSR)—can attenuate this process: lonely older adults participating in an 8-week MBSR program showed reduced activity of NF-κB (a key pro-inflammatory transcription factor) and improved *in vitro* immune responses following stimulation (e.g. increased IL-6 production when appropriately stimulated), indicating a restoration of immune competence in the face of stress (108, 109).

Another study in older adults with mild cognitive impairment found that regular mindful awareness practice significantly lowered high-sensitivity CRP levels over nine months, especially among females, suggesting durable reductions in systemic inflammation (110). In patients with end-stage renal disease—who typically experience high systemic stress and inflammation—mindfulness meditation reduced CRP and TNF-α, although IL-6 changes were not always significant, pointing to heterogeneity in effect by context or condition (108). Taken together, these findings indicate that psychosocial support and structured stress reduction can modulate inflammatory markers, preserve immune responsiveness, and may slow immunosenescence.

### 6.4 Integrated lifestyle approaches

Combining multiple lifestyle interventions (**Figure 2D**) produces synergistic benefits that go beyond what any single element might achieve. For example, in older adults, higher adherence to the Mediterranean diet combined with regular resistance training has been associated with a lower “immunological age,” characterized by reduced frequencies of senescent T cells and lower levels of pro-inflammatory cytokines, whereas interventions with exercise alone produce smaller or less durable improvements (111). Likewise, a recent trial of resistance training plus polyphenol supplementation among aging individuals demonstrated that the combo more effectively attenuated acute inflammatory responses (such as TNF-α and IFN-γ spikes post-exercise) when compared to resistance training without polyphenols (112).

Systematic reviews and network meta-analyses further support that exercise combined with dietary interventions (e.g. calorie restriction, high-fiber diet) yield better metabolic, body composition, and inflammation outcomes than either component in isolation – although many studies still stop short of deeply characterizing immune function at the cellular level (113). Large population-based analyses such as those from the UK Biobank show that persons who combine anti-inflammatory dietary patterns, sufficient physical activity, and healthy sleep exhibit delayed biological aging and reduced mortality risk (114).

Adequate sleep and stress management further enhance immune resilience: sleep quality amplification and stress reduction have been shown to improve anti-inflammatory cytokine profiles, help restore circadian-regulated immune cell trafficking, and reduce basal systemic inflammation. Microbiome-targeted nutrition—such as fiber-rich foods, fermented food inclusion, and perhaps polyphenol intake—helps maintain gut-immune homeostasis by promoting beneficial SCFA-producing taxa, preserving gut barrier integrity and limiting microbial translocation, which together help blunt inflammaging. Altogether, these integrated strategies represent a holistic framework for counteracting immunosenescence, reducing infection risk, and lowering burden of age-associated diseases (114).

Taken together, these integrated strategies represent a holistic approach to counteracting immunosenescence and inflammaging, promoting healthy aging and reducing susceptibility to infections, chronic inflammation, and age-associated diseases.

### 6.5 Personalized nutrition and lifestyle interventions

Personalized nutrition and lifestyle strategies (**Figure 2E**) are increasingly viewed as a translational pathway toward healthier immune aging. The integration of immunoprofiling and multi-omics tools (transcriptomics, metabolomics, microbiome sequencing, epigenetics) makes it possible to identify individual immune vulnerabilities—such as altered T-cell subsets, pro-inflammatory cytokine signatures, metabolic imbalances, or shifts in microbiome composition—and to design tailored interventions accordingly (115–118). In this framework, nutrition, exercise, sleep, and stress management are no longer generic recommendations but dynamic tools that can be adapted to the biological and lifestyle context of each person.

The growing convergence of systems biology with digital health monitoring further strengthens this approach. Wearable devices and continuous biomarker tracking enable feedback loops between intervention and outcome, allowing strategies to be adjusted in real time as individuals age. Early evidence suggests that such multi-domain interventions, when combined and personalized, can reduce inflammaging, preserve immune competence, and improve responsiveness to vaccines and infections (119, 120).

As research moves forward, the challenge lies in identifying the optimal combinations and timing of these interventions while ensuring feasibility and adherence in real-world settings. This precision lifestyle medicine paradigm provides not only a framework for mitigating immunosenescence but also a conceptual bridge to the broader vision of integrated, systems-level approaches that shape the future of immune aging research and clinical translation.

## 7 Conclusions and future directions

Rather than an inevitable decline, immune aging emerges as a process that can be reshaped. By uncovering the cellular and molecular mechanisms of immunosenescence and inflammaging, new opportunities arise to delay, redirect, or even partially reverse these trajectories. Diet, lifestyle, and emerging therapeutics are no longer just supportive measures but active switches to influence the pace and quality of immune aging.

Future directions call for integrating multi-omics profiling and multilayer network approaches to capture the dynamic interplay of immune, metabolic, and microbial systems across the lifespan. Immune aging should be reframed as a dynamic, multi-scale process that can be understood—and importantly—shaped. Translating mechanistic insights into real-world benefit will require two complementary pillars. First, systems biology coupled with longitudinal cohort studies: repeated, deep immune phenotyping across decades (multi-omics, functional assays, and clinical readouts) is essential to disentangle individual trajectories, identify early inflection points, and discover robust biomarkers of resilience versus decline. Second, multilayer network approaches that integrate immune, metabolic, and microbial layers will reveal the cross-talk and leverage points that single-axis studies miss.

Artificial intelligence (AI) may offer a powerful complement to systems biology in the quest for personalized strategies against immune aging. By integrating multi-omics data with insights from longitudinal cohort studies, AI can chart individual trajectories of immunosenescence and identify optimal intervention points. This vision requires secure data infrastructures, protection of privacy, and transparency in algorithmic design, but if achieved, AI could transform precision approaches to modulating immunosenescence and inflammaging.

Armed with trajectory maps and network-derived targets, the field can then design adaptive clinical trials that combine dietary, lifestyle, and pharmacological strategies to test timing, dose, and synergistic effects. The ultimate aim is not a one-size-fits-all remedy but trajectory-informed, personalized interventions that shift an individual’s immune course toward sustained resilience. By marrying longitudinal systems mapping with pragmatic, multi-modal interventions, we can move from describing immune decline to deliberately redirecting it—turning healthy longevity from aspiration into achievable practice.
